# Mesenchymal Stem Cells Ameliorate Th1-Induced Pre-Eclampsia-Like Symptoms in Mice via the Suppression of TNF-α Expression

**DOI:** 10.1371/journal.pone.0088036

**Published:** 2014-02-18

**Authors:** Liu Liu, Guangfeng Zhao, Hongye Fan, Xiaoyin Zhao, Pengfei Li, Zhiqun Wang, Yali Hu, Yayi Hou

**Affiliations:** 1 Immunology Lab, Medical School and State Key Laboratory of Pharmaceutical Biotechnology, Nanjing University, Nanjing, China; 2 The Affiliated Drum Tower Hospital of Nanjing University Medical School, Nanjing, China; 3 Jiangsu Key Laboratory of Molecular Medicine, Nanjing, China; Michigan State University, United States of America

## Abstract

Pre-eclampsia (PE) is thought to be a pregnancy-induced autoimmune disease. Despite several strategies carried out for targeting specific factors relevant to its pathogenesis, PE remains potentially fatal to some patients. Here, we reported a way to isolate mesenchymal stem cells (MSCs) from decidua. The MSCs not only exhibited differentiation and self-renewal capacities, they also possessed immunomodulatory functions and secreted some soluble mediators including IL-6, TGF-β, IDO, VEGF and COX-2. Most importantly, the MSCs were specifically provided with the ability to suppress T cells proliferation by IDO in response to inflammatory cytokine IFN-γ. Moreover, we developed a Th1 cell-induced PE mouse model which displayed a high level of pathogenesis factor TNF-α. Strikingly, MSCs-based therapy significantly ameliorated both clinical and histopathological severity of PE symptoms including decreasing the blood pressure and proteinuria, suppressing glomerulonephritis, protecting the feto-placental development. The therapy also reversed abnormal TNF-α expression in uterine and splenic lymphocytes. These data suggest that MSCs may ameliorate Th1-induced PE-like symptoms in mice via the suppression of TNF-α and MSCs-based therapy may provide a potential novel method for PE.

## Introduction

PE is a common obstetric syndrome affecting women in their first pregnancy and characterized by hypertension and proteinuria which appeared after 20 weeks of gestation. The etiology and pathogenesis of this syndrome are not fully understood. Much evidence has indicated that PE may be a pregnancy-induced autoimmune disease, as a consequence of the immune imbalance at the maternal-fetal interface [1]–[3]. In human pregnancy, there is a shift of cytokine production toward Th2-type immunity with predominance of IL-4 and IL-10 in stimulated peripheral blood mononuclear cells (PBMC) [4], thus, pregnancy is considered as a Th2 phenomenon. However, in PBMC derived from some women with PE, Th1 cytokines expression increased and Th2 cytokines expression decreased, implying the balance shifts toward Th1-type immunity[5]–[7]. Our previous data also showed the decreased percentages of Tc2 and Th2 cells and the increased ratios of Tc1/Tc2 in both decidua and maternal peripheral blood of PE patients [8]. Moreover, the increase of Th1-type cytokine TNF-α was thought to associate with PE [9]–[12]. It was reported that TNF-α was involved in the activation of autoantibody-mediated angiotensin receptor. Both TNF-α and angiotensin receptor autoantibody affected the production of soluble fms-like tyrosine-1 and soluble endoglin, which are two critical etiological factor in PE [13], [14]. So TNF-α has been speculated to contribute to pathogenesis of this disease.

Several strategies have been developed for targeting specific factors relevant to PE pathogenesis. VEGF121 administration in Sprague-Dawley rats with elevated sFlt-1 levels successfully ameliorated main features of the PE such as hypertension, proteinuria, and glomerular endotheliosis [15]. RmPlGF-2 treatment also significantly decreased the blood pressure in PE-like mouse with overexpression of sFlt-1[16]. Chronic treatment with superoxide dismutase-mimetic tempol throughout gestation significantly improved fetal growth and survival, and ameliorated pregnancy-induced increases in blood pressure and proteinuria in BPH/5 mouse [17]. However, none of these therapies were focused on the immune disorder of PE. Therefore, we hypothesize that develop a therapy to promote immune balance may be a reasonable approach for effective control of PE.

MSCs not only possess extensive self-renewal potential and the ability to modulate immunocyte activation [18], [19], but they are also easily expanded and stored in vitro. Under stress conditions, MSCs home to sites of injury where they participate in tissue repair and regeneration [20], [21] and modulate the function of immune cells. Our previous data also demonstrated the immunosuppressive and anti-inflammatory effects of MSCs in the treatment of several animal disease models including autoimmune diseases [22]. As a result of these unique qualities, MSCs may be an attractive candidate in stem cell-based strategy for PE.

In this study, we developed a way to isolate MSCs from decidua. The MSCs exhibited differentiation and self-renewal capacities and immunomodulatory functions including secreting soluble mediators IL-6, TGF-β, IDO, VEGF and COX-2. The MSCs also specifically suppressed T cell proliferation by IDO in response to IFN-γ. Moreover, we developed a Th1 cell-induced PE-like mice model as previous reported method [23]. Strikingly, we first found that MSCs-based therapy could ameliorate both clinical and histopathological severity of PE-like symptoms including the blood pressure and proteinuria, glomerulonephritis and feto-placental development. The MSCs-based therapy also reversed the abnormal TNF-α expression in uterine and splenic lymphocytes.

## Materials and Methods

All research involving human participants have been approved by the “Drum Tower Hospital ethics committee” and written informed consent was obtained from all subjects. All research involving animal was carried out strictly accordance with the recommendations in the Guide for the Care and Use of Laboratory Animals of the National Institutes of Health. All efforts were made to minimize suffering. The protocol was approved by the Committee on the Ethics of Animal Experiments of Drum Tower Hospital ethics committee.

### Isolation and culture of human MSCs

A umbilical cords derived MSCs isolating method which reported by our group were used here to isolate MSCs from deciduas [24]. Human decidua were obtained from the Department of Gynecology and Obstetrics, the Affiliated Drum Tower Hospital of Nanjing University Medical School from full-term caesarean section births. The deciduas tissue were collected from the maternal part of placenta. Decidua tissues were transferred to a sterile container in DMEM/F12 (Gibco) with antibiotics (penicillin 100 µg/ml, streptomycin 10 µg/ml; Invitrogen Life Sciences) and was diced into 1–2 mm^3^ fragments. The tissue was incubated in an enzyme cocktail (hyaluronidase 5 U/ml, collagenase 125 U/ml and dispase 50 U/ml; SIGMA) for 15 to 30 min with gentle agitation at 37°C. The cells were pelleted by low-speed centrifugation (250 g for 5 min), suspended in fresh medium and transferred to 6-well plates containing the DMEM/F12 along with 20% fetal bovine serum. Cells were incubated at 37°C in an incubator with 5% CO_2_ at saturating humidity. When cells reached 70%–80% confluency or when numerous colonies were observed, the cells were detached with 0.25% trypsin-EDTA (Invitrogen), the trypsin was inactivated with fresh media. After four cell passages, the surface antigens of the adherent cells were detected.

### Flow cytometry

The following human mAbs, purified or directly conjugated with FITC, PE or APC, were used in FACS® analysis: human anti-CD8, anti- IFN-γ, mouse anti-IL4, anti-CCR5, anti-CD69, anti-TNF-α, anti- IFN-γ as well as IgG and IgM isotype. For fluorescence measurements only, data from 10,000 single cell events were collected using a standard FACS calibur™ flow cytometer (Immunocytometry Systems; Becton Dickinson). Data were analyzed using CELL Quest™ (Becton Dickinson).

### Osteogenic and adipogenic differentiation

Cells were plated in six-well plates at a density of 1×10^4^ cells/cm^2^. Specific induction medium was added 24 h later. The osteogenic induction medium consisted of DMEM-LG supplemented with 10% FBS, 3.06 mg/ml β-glycerophosphate, 100 nmol/L dexamethasone, 10 nmol/L 1, 25-(OH)_2_ Vitamin D3 and 0.15 mmol/L ascorbic acid-2-phosphate. The adipogenic induction medium consisted of DMEM supplemented with 10% FBS, 1 µmol/L dexamethasone, 10 µg/mL insulin, 0.5 mmol/L isobutylmethylxanthine (IBMX), and 200 µmol/L indomethacin. Cells maintained in regular growth medium were regarded as the control. All the reagents for induction were purchased from Sigma. After 3 weeks of induction, the cells were stained using the alizarin red staining or oil red solution to detect the presence of calcium deposition in osteocytes or neutral lipid vacuoles in adipocytes, respectively.

### RT and QPCR analysis

Total RNA was extracted using Trizol Reagent (Invitrogen, Carlsbad, CA, USA) follow manufacturer's instructions. RNA concentrations were measured with the smartspec™ plus spectrophotometer (Bio-Rad, Hercules, CA, USA). RNA integrity was determined using formaldehyde denaturalization agarose gel electrophoresis. In order to quantify mRNA, total RNA (1 µg) was reverse-transcribed using Revert Aid™ First Strand cDNA Synthesis Kit (Fermentas). Subsequently, the expression level of possible target genes was determined by SYBR Green assays (Invitrogen) with an Applied BioSystems 7300 Sequence Detection System (Perkin-Elmer Applied Biosystems, Foster City, CA, USA). 1 µl of complementary DNA was used as the template for the following PCR. The reactions were incubated in a 96-well plate at 95°C for 5 min followed by 40 cycles of 95°C for 15 s, 60°C for 30 s, and 72°C for 30 s. All experiments were done in triplicate time. The levels of expression were calculated based on the PCR cycle number (Ct). Primer oligonucleotides were synthesized by Invitrogen and listed in Table 1.

**Table 1:** Primer information.

SANGER_NAME	Forward Primer (5′ - 3′)	Reverse Primer (5′- 3′)
HUMAN-TGF-β1	CAGCAACAATTCCTGGCGATAC	GCTAAGGCGAAAGCCCTCAAT
HUMAN -IL-6	TTCGGCAAATGTAGCATG	AATAGTGTCCTAACGCTCATAC
HUMAN- VEGF-A	AGGGCAGAATCATCACGAAGT	GCTGCGCTGATAGACATCCA
HUMAN- IDO	GATGTCCGTAAGGTCTTGCCA	TGCAGTCTCCATCACGAAATG
HUMAN- COX-2	CTGGCGCTCAGCCATACAG	CACCTCGGTTTTGACATGGGT
HUMAN-GAPDH	AGAAGGCTGGGGCTCATTTG	AGGGGCCATCCACAGTCTTC
MOUSE-TNF-α	TCGAGTGACAAGCCTGTAGC	CTCAGCCACTCCAGCTGCTC
MOUSE-IFN-γ	GTTTGAGGTCAACAACCCACAG	GCAGCGACTCCTTTTCCG
MOUSE-IL4	CATCCTGCTCTTCTTTCTCG	CCTTCTCCTGTGACCTCGTTC
MOUSE-GAPDH	AACGACCCCTTCATTGAC	TCCACGACATACTCAGCAC

### Western blotting and ELISA

Cells were lysed in a buffer containing 50 mM Tris-Cl pH 8.0, 150 mM NaCl, 0.02% NaN3, 0.1% SDS, 100 µg/ml phenylmethylsufonyl fluoride (PMSF), 1 µg/ml Aprotinin, 1% Triton. After centrifugation, cell lysates (40 µg/lane) were subjected to 12% SDS-PAGE and transferred onto polyvinylidene difluoride membranes (Millipore). The membranes were blocked for 1 h in PBST (10 mM Tris-HCl, pH 7.4, 150 mM NaCl, 0.05% Tween-20) containing 5% BSA. Protein bands were detected by the enhanced chemiluminescence (ECL) reaction (Kibbutz Beit Haemek, Israel). The antibodies against human IDO and COX-2 for western blot were from Santa Cruz biotechnology (San Diego, CA, USA).

Protein levels of IL-6, TGF-β and VEGF in cell supernatants were measured by enzyme-linked immunosorbent assay (ELISA). Cell growth media were collected 48 h after culture. Human precoated Elisa kits were used according to the instructions of the manufacturer (Beijing, China, DAKEWE, http://www.dakewe.net/).

### T cell proliferation and activation assay and mixed lymphocyte reaction

PBMC were obtained from healthy adult donors after written, informed consent. PBMC were isolated from heparinized venous blood by Ficoll-Paque gradient centrifugation. T cells were purified by anti-CD3 mAb-conjugated microbeads (Miltenyi Biotec, Bergisch Gladbach, Germany) according to the manufacturer's instructions. For T cell proliferation, purified T cells were stimulated with Phytohaemagglutinin (PHA; 5 µg/ml; Sigma) in the presence or absence of the MSCs. after 72 hours, T cells were harvested, and their proliferation was measured using [^3^H] thymidine incorporation. For T cell activation, T cells were harvested after 24 hours coculture and CD8^+^ cells were analyzed for the expressions of CD25, CD69 and intracellular IFN-γ production. For mixed lymphocyte reaction, purified T cells were seeded in 96-well flat-bottomed plates at 10^5^ cells with MSCs (MSCs: T cells = 1∶5, 1∶10, 1∶20 and 1∶50) in a final volume of 200 µl 1640 medium/well. PHA (5 µg/ml) was used to induce the proliferation of T cells. The proliferation of T cell was determined by [^3^H] incorporation assay. The IDO (1-MT; 1 mM) and COX-2 (indomethacin; 5 µM) inhibitor was from Sigma and TGF-β (10 µg/ml) and IL-6 (10 µg/ml) neutralizing Ab was from Ebioscience.

### Development of PE-like mouse model

Specific pathogen-free 4–6 weeks-old C57BL/6 (male) and BALB/c (female) mice were purchased from Model Animal Research Center (MARC) of Nanjing University, PR China. Mice were maintained under specific pathogen-free conditions in a temperature-controlled room (24°C), on a 12-h/12-h light and dark cycle. Standard laboratory pelleted formula and tap water were provided. Each group of mice was housed in separate rack-mounted wire cages. All the cages were placed in the same shelf. A quick death procedure by cervical dislocation was uniformly performed in all animals. The experiments were conducted according to institutional animal ethics guidelines. All efforts were made to minimize suffering.

Two-month-old female BALB/c mice were mated with three-month-old male C57BL/6 ones. The day of finding vaginal plug was considered as day 0.5 of pregnancy, and plugged females were removed from breeding cages. Spleens from female BALB/c mice were crushed in culture dishes with RPMI 1640 medium containing 10% FCS. Mononuclear cells were isolated by density gradient and activated by incubation with anti-CD3 mAb (eBiosciences) at 3 µg/ml for 20 min. The cells (1.5×10^6^ cells/ml) were placed in culture media and incubated for an additional 30 h in an atmosphere containing 5% CO_2_ at 37°C. The culture media consisted of RPMI 1640 containing recombinant murine IL-2 (2 ng/ml) and IL-12 (8 ng/ml, Peprotech). Cell viability and cell number were determined by the trypan-blue exclusion assay. After 48 h culture, cells were washed twice with PBS solution, adjusted to a concentration of 10^7^ cells/100 µl with sterile PBS, and injected into the recipients (i.v.). 10^7^ activated Th1 cells were injected into the recipients at day 10.5 and day 12.5, respectively. MSCs suspended in 100 µl of PBS (10^6^ cells/100 µl) were injected (i.v.) into mice at day 11.5 and day 13.5, respectively. The mice were sacrificed at day 14.5. The fetal rejection rate, placental weights, fetal weights and fetal numbers were recorded after mice were sacrificed at gestated day (DG) 14.5.

Systolic blood pressure was measured in calm, warmed and restrained mice by the tail-cuff method at DG 14. As reported previously [25], a 17 mm tail cuff was applied and a pulsetransmitter (AD Instrument, NIBPController) was applied to the tail. The animals were restrained for 5 min using a plastic holder designed for mice in a temperature controlled chamber (30°C). Restrained mice were transferred into another chamber maintained at 30°C for measurements. The average of 10 recordings taken at 1 s intervals without movement artifacts were considered as the systolic blood pressure. Urine samples were analyzed for proteinuria using pyrogallol phenol red kit (DiaSys Diagnostic,Germany) in automatic biochemical analyzer (Hitachi 7600-020, Japan).

### Collection of uterine and splenic lymphocytes

Mice were killed by cervical dislocation and spleens were removed aseptically. Splenic single-cell suspensions were prepared by teasing with sterile forceps, digested with 1 mg/ml collagenase D for 30 min at 37°C, and then filtrated through the 100-mesh and 200-mesh cell strainer. The supernatants were collected and loaded on Ficoll density gradient to purify the splenic lymphocyte population. Uterine tissue was trimmed into 1-mm pieces and enzymatically digested for 20 minutes, using vigorous shaking, with 1.5 mg type I DNAse and 24 mg type IV Colagenase present in 15 ml RPMI-1640 medium. This procedure was repeated thrice. After an additional 5-minute incubation at room temperature without shaking, the supernatants were collected and loaded on Ficoll density gradient to purify the lymphocyte population.

### Statistical analysis

Results were presented as mean±S.E.M. Statistical analysis was performed with ANOVA followed by the Newman-Keuls test for multiple comparisons between different study groups. A value of p<0.05 was considered statistically significant.

## Results

### Isolation and characterization of MSCs-derived from human decidua

**Figure 1:**
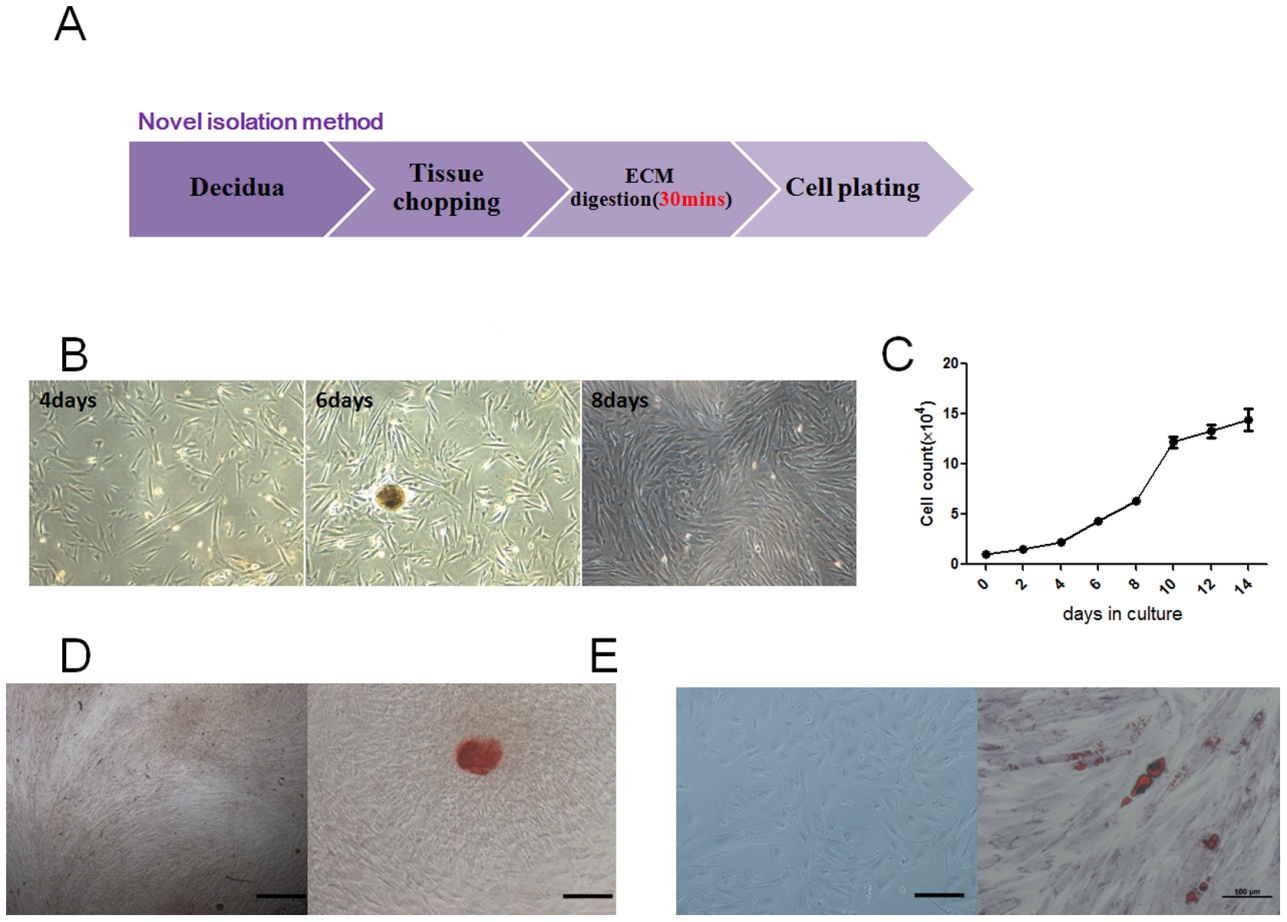
Isolation and characterization of MSCs derived from human decidua. (A): Schematic description of the way to isolate MSCs. (B): Morphology of MSCs isolate after 4 day, 6 day and 8 day (20X). (C) Cell growth curve of MSCs. UCMSCs of passage 2 were plated in 24-well plates in DMEM/F12 supplemented with 10% FBS at a density of 5×10^3^ cells/well. The cells were detached with 0.25% trypsin-EDTA every 2 days and cell counting until 14 day. Bars show the mean. n = 3. (D) Osteogenic differentiation was assayed by the alizarin red staining. No mineralized matrix formation was found in UCMSCs maintained in regular growth medium. Osteogenic differentiation was evidenced by the formation of mineralized matrix in UCMSCs after osteogeneic induction. (10X). Scale bars, 100 µm. (E) Adipogenic differentiation was detected by oil red O staining. No lipid vacuoles were found in in UCMSCs maintained in the regular medium. Adipogenic differentiation was evidenced by the formation of lipid vacuoles by oil-red O staining in UCMSCs after adipogenic induction. (10X) Scale bars, 100 µm. All data are representative of three independent experiments.

A novel way was developed for isolating MSCs from human decidua here.In general, an enzyme cocktail (hyaluronidase 5 U/ml, collagenase 125 U/ml and dispase 50 U/ml; Sigma) instead of collagenase was used here to digest the fragments of decidua. After washing twice, large pieces of tissue in the digested mixture did not need to be removed and the digested mixture also did not need to pass through a 100 µm filter to obtain single cell suspensions [Fig. 1A]. Adherent cells with fibroblastic morphology could be observed as early as 24 hours. The cells formed a monolayer of homogenous bipolar spindle-like cells with a whirl pool like array within 1 week [Fig. 1B]. Then, MSCs were serially passaged to examine their expansion potential and determined to expand readily for up to 2 weeks [Fig. 1C]. After 3 cell passages, the adherent cells were symmetric with phenotypic surface antigens, which is positive for CD105, CD73, CD90, HLA-ABC, CD29, CD44 and negative for HLA-DR, CD19, CD11b, CD14, CD34, CD31 (date not show). In addition, our obtained MSCs possessed the abilities of osteogenic differentiation [Fig. 1D] and adipogenic differentiation [Fig. 1E], which detected by the calcification of the matrix (alizarin red staining) and oil red O staining, respectively.

### MSCs are determined to have suppressive ability on T cells

**Figure 2:**
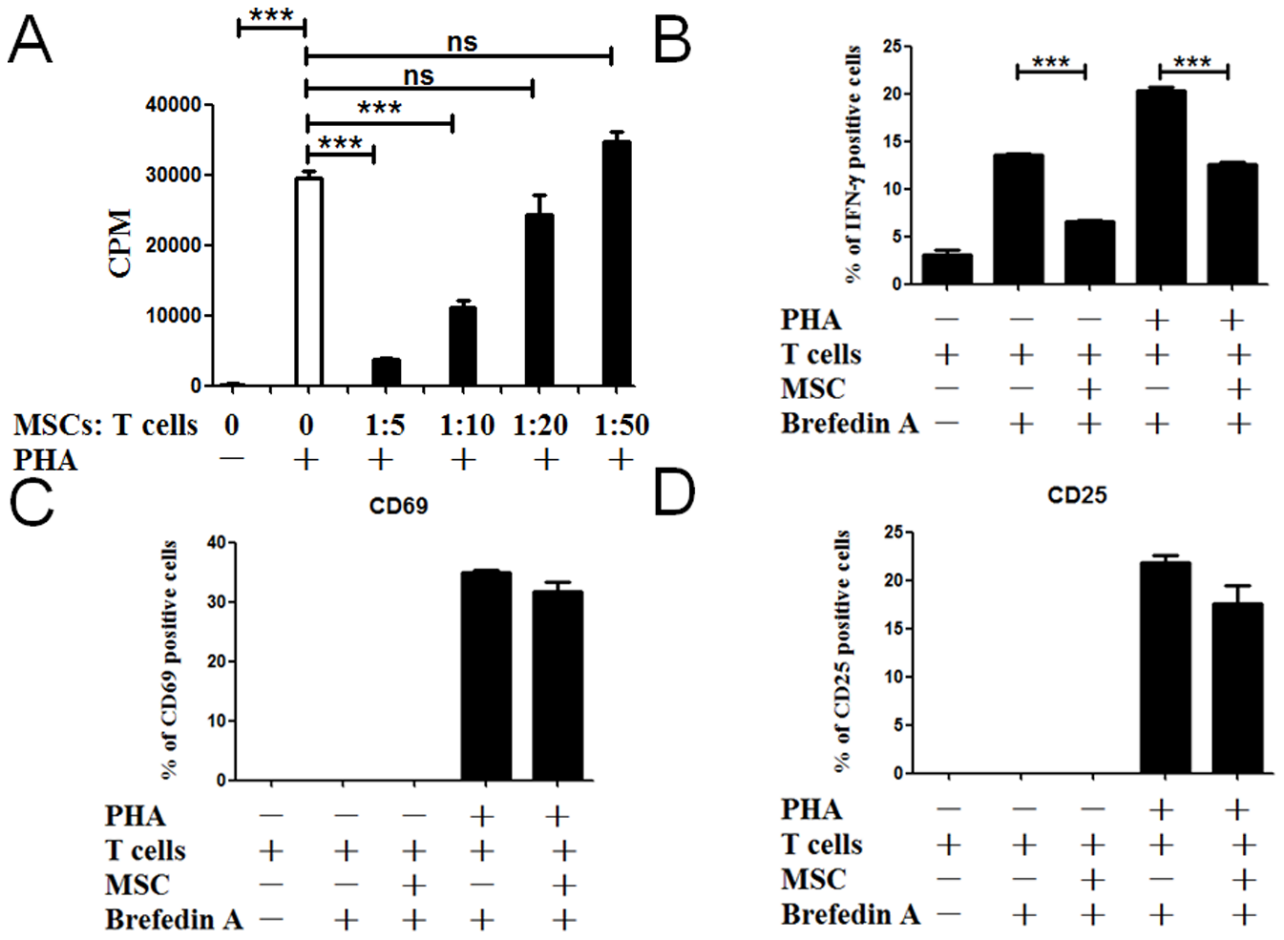
MSCs are capable of suppressing T cells proliferation. T cells were cocultured with MSCs. All T cells were activated with PHA/IL-2 except control. Different MSCs: T ratios (1∶5, 1∶10, 1∶20 and 1∶50) were used to perform the MLR. T-cell proliferation was assessed by [^3^H] thymidine incorporation assay after 72 h of culture (A). n = 4. When it says NS, they are being compared to the grey column (MSC 0 PHA +). (B–D): T cells activated with or without PHA/IL-2 were cultured with UCMSCs at MSCs: T ratios (1∶10). After 24 hours, CD8^+^ cells were analyzed for intracellular IFN-γ and for the expression of activation molecules using anti-CD25 or anti-CD69 monoclonal antibodies. For IFN-γ staining, T cells were treated with Brefeldin A for the last 4 hours of cultures. Cells were permeabilized and the proportion of CD8^+^/IFN-γ^+^, CD8^+^/CD69^+^ and CD8^+^/CD25^+^ T cells was quantified. n = 4. All data are representative of three independent experiments. ***, p<0.001.

It is well known that MSCs are able to suppress T-cells activation. To determine whether the MSCs obtained by our new way also have similar effects, T cell proliferation was measured and the results demonstrated that the MSCs inhibited the proliferation of activated T cells [Fig. 2A]. In addition, CD8^+^ cells were analyzed for the expressions of CD25, CD69 and intracellular IFN-γ production. The results showed that MSCs impaired IFN-γ production [Fig. 2B] and slightly affected the expression of CD25 and CD69 [Fig. 2C, D].

### MSCs-secreted soluble mediators is involved in the inhibition of T cells

**Figure 3:**
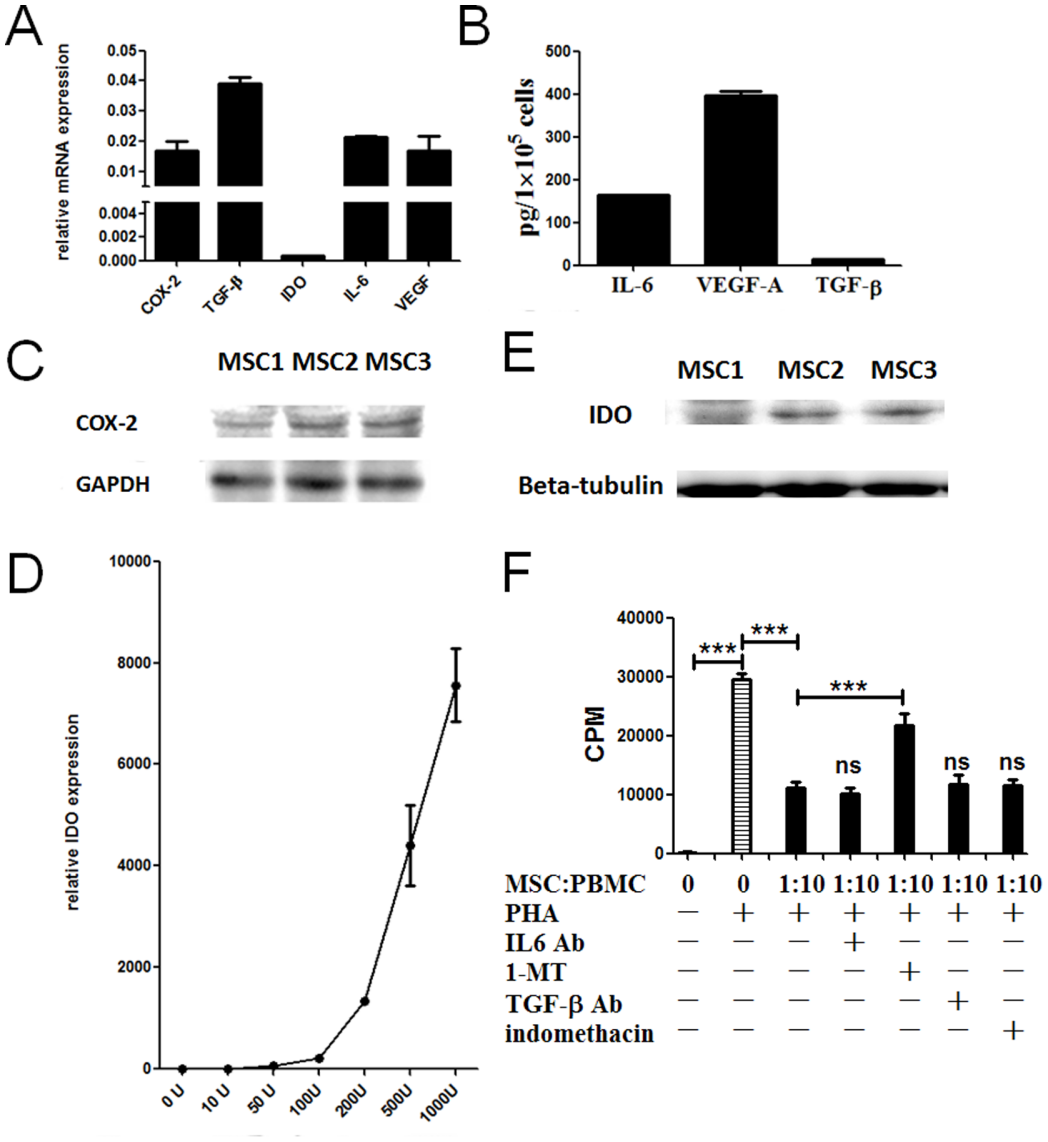
MSCs Secrete Soluble mediators involved in MSCs-mediated inhibition of T cells. (A): QPCR analysis of the expression of IL-6, TGF-β, IDO, VEGF and COX-2. Relative to GAPDH. n = 3 (B): The protein level of IL-6, VEGF-A and TGF-β expressions were detected by ELISA 48 h after Culture. (C): The protein level of COX-2 by western blot 48 h after culture. (D): MSCs were treated with indicated concentrations (0 u, 10 u, 50 u, 100 u, 200 u, 500 u and 1000 u pre millilitre) of IFN-γ for 24 h and then cells were harvested and total RNA was extracted. Quantitative mRNA expression was measured as described before. Data are shown as mean + SEM of n = 3 replicates and are representative of three independent experiments with three samples. (E): The protein level of IDO by western blot treated with 1000 u pre milliliter IFN-γ for 72 h. (F): PHA/IL-2-activated T cells were also cultured for 72 h with at MSCs: T ratios(1∶10) in the presence or absence of neutralizing Ab for TGF-β (10 µg/ml) and IL-6(10 µg/ml), antagonists for COX-2 (indomethacin; 5 µM) and IDO(1-MT; 1 mM). n = 4. All data are representative of three independent experiments. ***, p<0.001.

It is reported that bone marrow-drived MSCs secreted several soluble factors such as IL-6, TGF-β, IDO, VEGF and COX-2 to exert the immunosuppressive properties. To determine whether the MSCs also express these factors, mRNA expression for IL-6, TGF-β, IDO, VEGF and COX-2 [Fig. 3A] and protein level of IL-6, TGF-β, VEGF and COX-2 were detected [Fig. 3B–C]. The results showed that the MSCs secreted significant amounts of VEGF, IL6 and COX-2, but produced only minimal amounts of TGF-β. In addition, the basal expression of IDO in the MSCs is very low, while a substantial amount mRNA and protein of IDO can be induced by IFN-γ [Fig. 3D–E]. MSC1, MSC2 and MSC3 indicate that the MSCs were derived from three human decidua, respectively. Their relevant clinical details are shown in Table 2. To further investigate which of soluble factors plays role in the inhibition of T cells, anti-IL-6 and anti-TGF-β neutralizing Ab, 1-MT (an inhibitor of IDO enzymatic activity) and indomethacin (an inhibitor of PGE2 enzymatic activity) were added to the cultures, respectively. The results showed that just 1-MT significantly reversed the inhibition of MSCs on T cell proliferation [Fig. 3F]. This suggests that IDO may contribute, in part, to MSCs-mediated suppression of T cells.

**Table 2:** Clinical characteristics of the study population.

	MSC1	MSC2	MSC3
Age, years	28	27	27
Gestational age at delivery, week	38	39	38
primiparae	Yes	Yes	No
Body mass index, kg/m^2^	27.1	28.4	27.9
Systolic blood pressure, mm Hg	119	115	121
Diastolic blood pressure, mm Hg	81	83	79
Proteinuria, mg/24 h	0	0	0
Alanine aminotransferase, U/L	37	30.5	31.2
Blood urea nitrogen, mmol/L	3.8	3.2	4.1
Platelet, ×10^9^	192.0	187.3	197.2
Birth weight, g	3412.3	3212.7	3613.1
Placenta weight, g	520.1	521.4	530.9

### MSCs-based therapy ameliorates symptoms of Th1-induced PE-like mice

**Figure 4:**
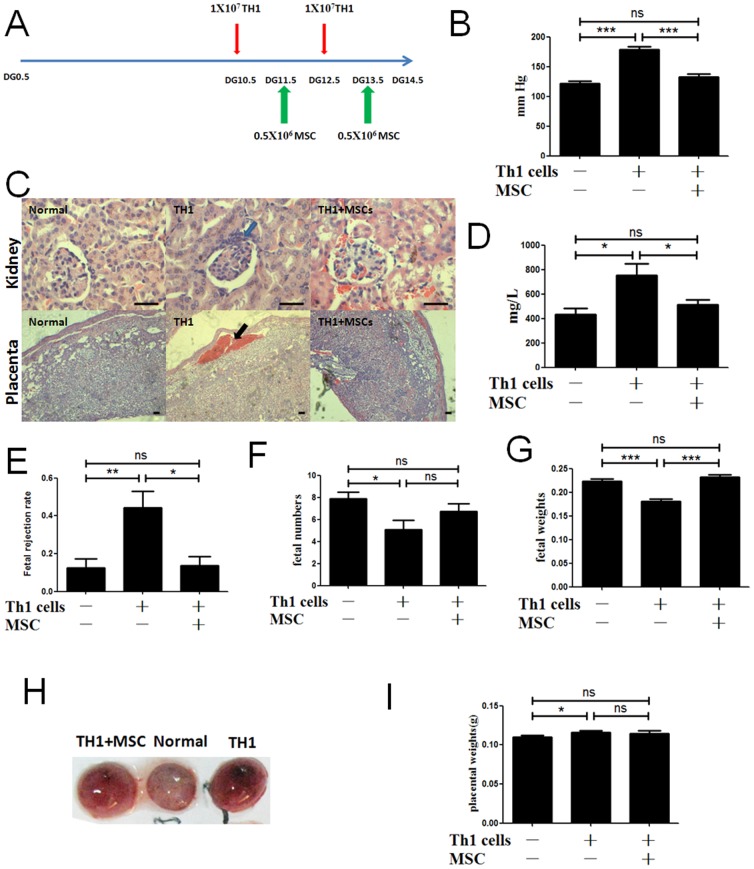
MSCs -based therapy ameliorates TH1-induced PE in mice. (A): Scheme for the experimental course of PE model induced by activated Th1 cells and MSCs-based therapy. Blood pressure (B) was detected on day on DG14. (C): Histopathological analysis of kidney and placenta after sacrificed. Representative examples of abnormal cell distribution as well as cell clusters in the glomerulum of pregnant cell recipients (blue arrow); while normal pregnant animals show no pathological signs in the kidneys as representatively. Moreover, fibrosis and glomerular disorganization could also be observed in animals with PE-like signs. Animals that received Th1 activated cells and developed PE show large areas of massive haemorrhage (blood) in placenta (black arrow), MSCs infusion reversed the condition of PE. The objective lens used for the pictures were: for kidney, (40X); for placenta, (10X). Scale bars, 50 µm. Proteinuria (D) was detected on day on DG14. (E) The fetal rejection rate in pregnant mice after PBS treatment (n = 11) or transfer of activated Th1-like cells (n = 8) or transfer of activated Th1-like cells and MSCs (n = 5) as calculated from the ratio of rejected fetuses to total implantation sites. Fetal numbers (F), fetal weights (G), placental weights (I), was detected after the mouse were executed. (H): Representative picture of placentas from pregnant mouse. After transfer of activated Th1-like cells or transfer of activated Th1-like cells, some placenta showed placental bleeding. All data are representative of three independent experiments. *, p<0.05, **, p<0.01 and ***, p<0.001.

We next explored the potential therapeutic effects of MSCs infusion in Th1-induced PE model [Fig. 4A]. The results showed that Th1-induced PE mouse model was successfully developed and the mice were characterized by increased blood pressure, glomerulonephritis and proteinuria [Fig. 4B, C, D]. Moreover, transfer of Th1-like activated cells provoked an increased fetal rejection rate[Fig. 4E]. In addition, decreased survival, impaired feto-placental units, lower fetal numbers [Fig. 4F], fetal weights[Fig. 4G] and massive placental haemorrhage[Fig. 4H] were observed in Th1-induced PE mouse model, although, there is a slight increase in the placental weight after transfer of Th1-like activated cells[Fig. 4I]. Histological studies revealed fibrosis, glomerular disorganization and large areas of massive haemorrhage in placenta in Th1-induced PE mouse model [Fig. 4C]. Strikingly, MSCs transplantation ameliorate symptoms of Th1-induced PE mice and benefit feto-placental development including increased fetal weights and fetal numbers [Fig. 4B–I].

### Therapeutic effect of MSCs mainly depended on the decrease of TNF-α

**Figure 5:**
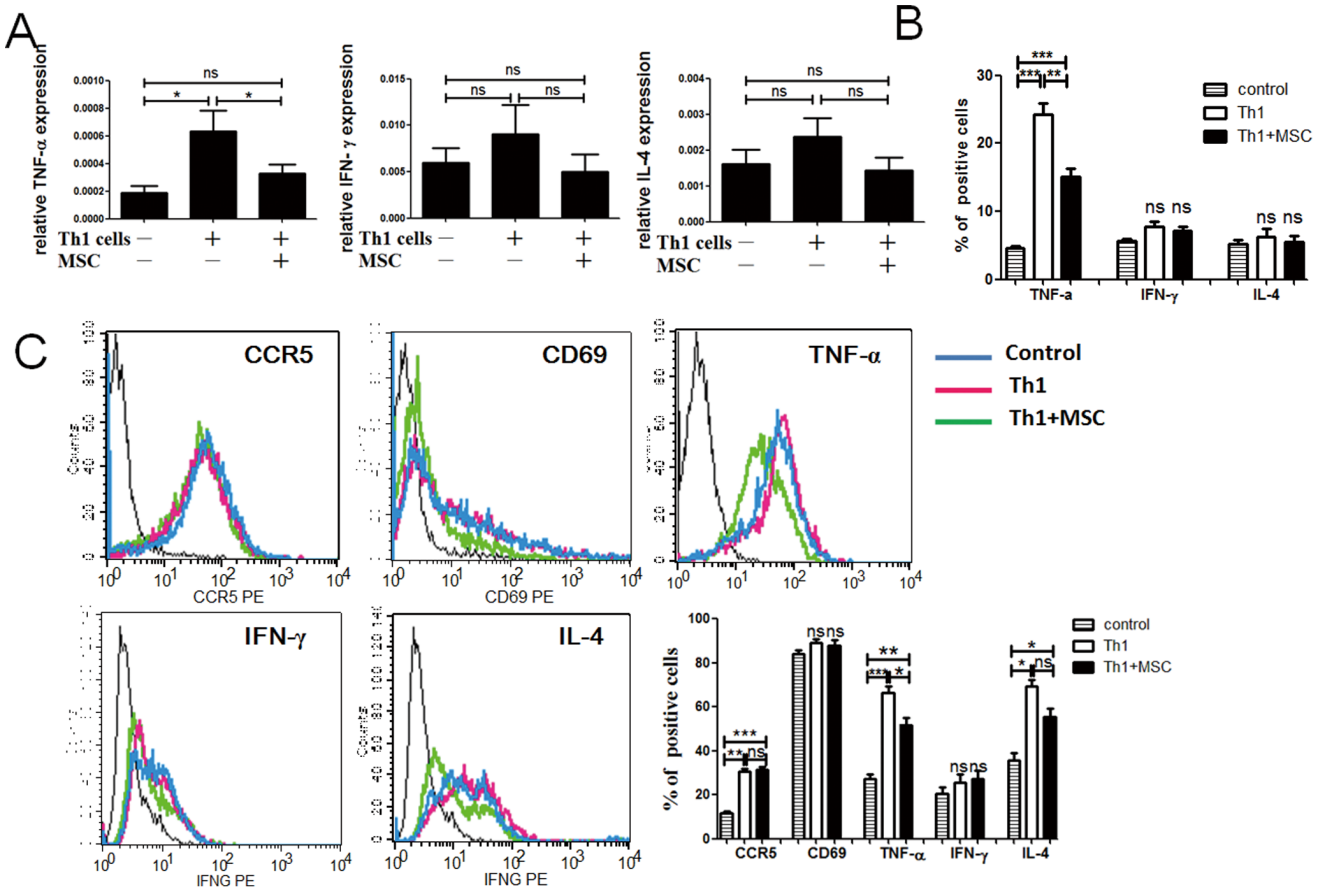
MSCs -based therapy reverse the cytokine abnormal in PE uterine and splenic lymphocytes. (A): QPCR analysis of the expression of TNF-α, IFN-γ and IL-4 in uterine tissue. Relative to GAPDH. (B): An illustration of the percentage of uterine lymphocytes positive for TNF-α, IFN-γ and IL-4 in pregnant mice that received PBS (n = 5) or activated Th1-like cells(n = 5) or activated Th1-like cells and MSCs(n = 5), as analyzed by flow cytometry. (C): Spleen lymphocytes were analyzed for the expression of activation molecules using anti-CD69 or anti-CCR5 monoclonal antibodies or analyzed for the expression of intracellular IFN-γ, IL-4 and TNF-α. Spleen lymphocytes were treated with PMA for the last 4 hours of cultures before the analysis. All data are representative of three independent experiments. *, p<0.05, **, p<0.01 and ***, p<0.001.

Compared with normal pregnancy mouse, Th1-induced PE mice showed aberrant expression levels of TNF-α in uterine and TNF-α and IL-4 in splenic lymphocytes, but no obvious difference in the expression of the IFN-γ and IL-4 in uterine orCCR5, CD69 and IFN-γ in splenic lymphocytes [Fig. 5A, B, C]. Importantly, MSCs significantly attenuated TNF-α expression in uterine and splenic lymphocytes [Fig. 5B–C]. These findings suggest that MSCs may ameliorate Th1-induced PE-like mice mainly via the suppression of TNF-α.

## Discussion

We developed a way to isolate MSCs from decidua. we previews reported a novel way to isolate MSCs from umibilical cords[24]. In this research, we test the efficiency of using our method to isolate MSC from decidua. Our results show that the method we developed is also high efficiency to isolate MSCs from deciduas. Most importantly, MSCs isolated using our method shared the same feature (capable of differentiating, self-renewal and immunosuppressive properties) with bone marrow-derived MSCs as reported.

MSCs are multipotent cells capable of differentiating and amplification in vitro and in vivo to different MSCs lineages, including adipose, bone, cartilage and myelosupportive stromal [26], [27]. On the other hand, MSCs also are low immunogenic cells and allogenic MSCs are not rejected by immune system. Recently, several studies from our and other groups[22], [28] have reported that treatment with human bone marrow-, adipose- or umbilical cords- derived MSCs exhibited early efficacy in attenuating the progression of several experimental inflammatory diseases in murine models, including experimental arthritis[28], colitis [29], [30], and autoimmune encephalomyelitis[31]. There are two reasons why those experiments are reasonable. First, MSCs are low immunogenicity cells. Base on our and other groups results, human MSCs do not rejected by mouse immune system, instead, those MSCs will home to sites of injury and affect as a immunosuppressive cells which make those cell have the protential way in injury site targeting and anti-inflammatory effect. Secondly, the finally purpose of those research are to provide a possible way to use human MSCs to cure human disease. Therefore, using human MSCs can help us to understand the role of human MSCs in human disease more clearly.

Some studies have indicated that PE may be a pregnancy-induced autoimmune disease. Decidual lymphocytes and PBMCs from patients with PE synthesize high levels of Th1 cytokines, eg, IL-1, IL-2, and IFN-γ. However, the secretion of the Th2 cytokines IL-10 and IL-5 is decreased. The circulating levels of TNF-α and IL-6, which are already more elevated in healthy pregnant women compared with non-pregnant women, are further raised in patients with PE [32]. Besides, a variety of animal models of PE also confirmed the correlation of abnormal immune system and PE including nitric oxide related model [33], Th1-type cytokines model [23] and renin angiotensin related model [1]. Published evidence indicates that Th1 cytokines can directly damage organs and destroy vessels which are the main cause of PE [23]. Moreover, the adoptive transfer of activated Th1-like cells also primed the host immune system and the activated immune cells primed to paternal antigens. On the other hand, TNF-α plays an important role in the pathogenesis of this PE-like model. Many authors have pointed out TNF-α as a key player in the etiology of PE [34]–[36]. This is supported by our data that very high amounts of TNF-α expression can be observed at the maternal-fetal interface and spleen of mice with PE-like symptoms. Therefore, promoting the immune balance and decreasing Th1 proinflammatory cytokine like TNF-α in maternal-fetal interface may be the key to effective control of PE.

An interesting character of MSCs is its immunosuppressive properties. The production of IDO by MSCs contributes to the immunosuppressive effect of MSCs [37], [38]. Roland Meisel suggests that IDO is the key players involved in the inhibition of T cell proliferation by MSCs[39]. Beside the ability of inhibiting the proliferation of immune cells, MSCs also reverse the expression of proinflammatory cytokine like IL-1β, TNF-α, IFN-γ in vitro and vivo. Decreased levels of TNF-α were observed after co-culture of hMSCs with anti-CD3/CD28-activated peripheral blood mononuclear cells (hPBMCs)[40]. Systemic infusion with MSCs also mediated a down-regulation of proinflammatory (TNF-α and IFN-γ) in several animal model including endotoxin-induced acute lung injury mice model, experimental autoimmune encephalomyelitis (EAE) and ischemia-reperfusion-induced severe acute renal failure model [41]–[43]. It should be noted that MSCs mostly mediated a down-regulation of Th1 proinflammatory cytokine. Therefore, MSCs infusion may be a reasonable approach for effective control of PE to develop a therapy to promote immune balance.

Decidua is the maternal-fetal interface where immunocyte interact with other support cells. We use MSC dervied from decidua instead of umibilical cords because we also want to further understand the protential mechanism underling. Our research already reported the abnormal of decidua-derived MSCs in PE[24] and the possiblility of MSCs function affect by maternal-fetal interface abnormal[44]. Therefore, infusion normal MSCs into PE might be a way to compensate the abnormal MSCs function in vivo. Our result not only provide a protential therapy to PE, it may also reavel the PE mechanism underling: the abnormal of MSCs in decidua lost the immunosuppressive ability to immunocyte, infusion normal MSCs into PE might be a way to compensate the abnormal MSCs function in vivo.

Indeed, there are two limitation of this study. Although many of mice models have features of preeclampsia, they are still poor overall models of the human disease and limited due to lack of reproducibility and because they do not include the complete spectrum of pathophysiological changes associated with preeclampsia. Some big animal model maybe need for further investigation. we also need to investigate the other possible mechanism for the treatment affect of MSCs since MSCs is a multifunctional cell. MSCs may affect this disease not only by ameliorate the immune system but also by promote angiogenesis[45], since MSCs also can secrete factors like VEGF and PDGF. A further research is needed to investigate the affect of MSCs on other PE model which is not induced by immune cells.

## Conclusions

In this study we develop a way to isolate MSCs from decidua. We found that MSCs-based therapy significantly ameliorate symptoms of Th1 cell-induced PE-like mouse model including decreasing the blood pressure and proteinuria, suppressing glomerulonephritis, protecting the feto-placental development. The therapy also reversed abnormal TNF-α expression in uterine and splenic lymphocytes. These data suggest that MSCs may play important roles in maternal-fetal interface immune balance. MSCs-based therapy may provide a potential novel therapy strategy for PE.
